# Enhanced Hemocompatibility of Silver Nanoparticles Using the Photocatalytic Properties of Titanium Dioxide

**DOI:** 10.3389/fbioe.2022.855471

**Published:** 2022-02-17

**Authors:** Xiao Chen, Sheng Dai, Luying Liu, Peng Liu, Peng Ye, Yuzhen Liao, Ansha Zhao, Ping Yang, Nan Huang, Jiang Chen

**Affiliations:** Key Laboratory for Advanced Technologies of Materials, Institute of Biomaterials and Surface Engineering, Ministry of Education, Southwest Jiaotong University, Chengdu, China

**Keywords:** Silver nanoparticles (AgNPs), Titanium dioxide (TiO_2_), hemocompatibility, antibacterial ability, UV treatment

## Abstract

Silver nanoparticles (AgNPs) are widely used because of their excellent antimicrobial properties. However, the poor hemocompatibility limits the application of AgNPs in blood contact materials. General approaches to improve the hemocompatibility of AgNPs-containing surfaces are to construct barrier layers or co-immobilize anticoagulant biomolecules. But such modification strategies are often cumbersome to prepare and have limited applications. Therefore, this study proposes a simple UV-photo-functionalization strategy to improve the hemocompatibility of AgNPs. We loaded AgNPs onto titanium dioxide (TiO_2_) nanoparticles to form a composite nanoparticles (Ag@TiO_2_NPs). Then, UV treatment was performed to the Ag@TiO_2_NPs, utilizing the diffusible photo-induced anticoagulant properties of TiO_2_ nanoparticles to enhance the hemocompatibility of AgNPs. After being deposited onto the PU surface, the photo-functionalized Ag@TiO_2_NPs coating showed excellent antibacterial properties against both Gram-positive/Gram-negative bacteria. Besides, *In vitro* and *ex-vivo* experiments demonstrated that the photo-functionalized Ag@TiO_2_NPs coating had desirable hemocompatibility. This modification strategy can provide a new solution idea to improve the hemocompatibility of metal nanoparticles.

## 1 Introduction

Silver has historically been a commonly used antibacterial material. When silver is oxidized, the free Ag^+^ released can act as an antibacterial/sterilizing agent by damaging the cell walls of bacteria and entering the bacteria to disrupt their metabolism and proliferation (Möhler et al., 2018). In the new century, various silver nanoparticles (AgNPs) have been developed and widely used in response to new needs. Due to their high specific surface area, AgNPs can release Ag^+^ efficiently and stably. Also, AgNPs with nanometer size can be directly uptaken by bacteria and thus combine with thiol-containing subcellular structures to kill bacteria synergistically with Ag^+^ (Zheng et al., 2018).

Due to their broad-spectrum and efficient antibacterial properties, AgNPs have been used in various medical devices and medical materials, such as burn dressing, catheter, and bone cement (Chaloupka et al., 2010). However, reports of AgNPs in blood contact devices are few because of the controversy of AgNPs’ hemocompatibility. Many studies have shown that AgNPs can cause adverse hematological events such as platelet adhesion and thrombosis when exposed to blood (Huang et al., 2016; Tran et al., 2022). This deficiency undoubtedly limits the use of AgNPs in blood-contact devices with antimicrobial needs, such as extracorporeal circuits and indwelling medical devices.

Researchers have tried many methods to improve the hemocompatibility of AgNPs. These approaches can be roughly classified into two categories. One is to construct a barrier layer to avoid direct contact between AgNPs and blood cells by utilizing biopolymer coatings or hydrogels (Fischer et al., 2015; Marulasiddeshwara et al., 2017). The other category involves co-immobilizing AgNPs with anticoagulant biomolecules to enhance hemocompatibility (Le Thi et al., 2017; Wu et al., 2020). However, both strategies are often cumbersome to prepare and do not address the hemocompatibility issue of AgNPs themselves. When the protective layer fails or AgNPs detach from the anticoagulant molecules, the AgNPs will face adverse hematological events again. Therefore, it is important to find a convenient and reliable improvement method to solve the hemocompatibility problem of AgNPs themselves.

TiO_2_ owns excellent biosafety and has a wide range of applications in the medical field (Jafari et al., 2020). Due to its unique photocatalytic activity, when TiO_2_ is subjected to UV irradiation, free radicals are generated, which can interact with the surrounding environment, thus making TiO_2_ exhibit biological activity (Ziental et al., 2020). Therefore, this photo-induced bioactivity of TiO_2_ has become a hot research topic in the last decade. The preliminary study of our team found that TiO_2_ after UV irradiation can acquire excellent anticoagulant properties. The changes in the physicochemical properties of the TiO_2_ surface, which are triggered by the TiO_2_ photo-generated free radicals, are thought to be responsible for this photo-induced anticoagulant properties (Chen et al., 2014). More importantly, we further revealed that this photo-induced anticoagulant properties could spread to the surface of silicon adjacent to TiO_2_ due to the diffusion effect of free radicals (Chen et al., 2015).

The mentioned understanding of AgNPs and TiO_2_ inspired us that it is possible to improve the hemocompatibility of AgNPs by utilizing the diffusible photo-induced anticoagulant properties of TiO_2_. To realize this idea, in this study, we firstly performed a short time of UV irradiation in seconds, using the TiO_2_’s photocatalytic reduction property to load AgNPs onto TiO_2_ nanoparticles (TiO_2_NPs) (Chen et al., 2021). Subsequently, we performed a one-hour UV irradiation to activate the photo-induced anticoagulant properties of TiO_2_. By the diffusion effect of the photo-induced anticoagulant properties, the hemocompatibility of AgNPs loaded on the TiO_2_NPs nanoparticles was enhanced. Finally, we obtained the photo-functionalized composite nanoparticles (UV-Ag@TiO_2_NPs) with anticoagulation and antibacterial properties.

In this study, microscopic appearance and elemental analysis by transmission electron microscopy (TEM) and energy dispersive spectroscopy (EDS) were used to examine the preparation of Ag@TiO_2_NPs. Water contact angle (WCA), photocatalytic degradation of methylene blue and silver ion release experiments were used to characterize the physicochemical properties of nanoparticles coating. Gram-positive *Staphylococcus aureus* and Gram-negative *Pseudomonas aeruginosa* were used to examine the antibacterial ability of photo-functionalized composite nanoparticles. *In vitro* platelet adhesion assay and *ex-vivo* antithrombogenicity test were used to examine the hemocompatibility of the photo-functionalized composite nanoparticles.

## 2 Materials and Methods

### 2.1 Preparation of Ag@TiO_2_NPs

**FIGURE 1:**
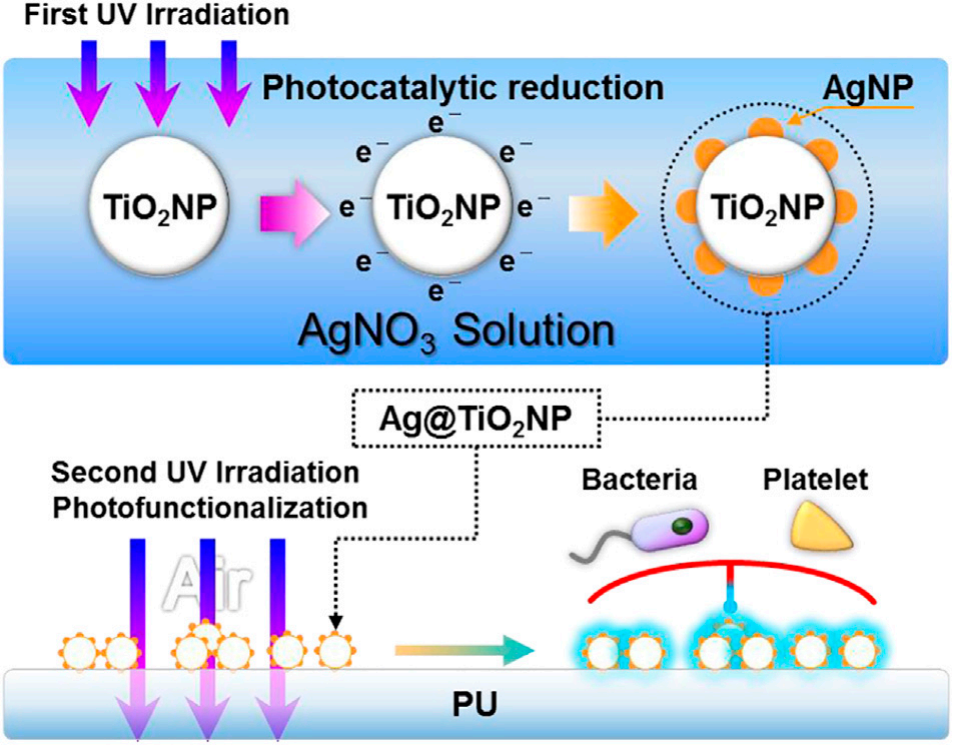
Schematic diagram of the preparation and photo-functionalization of composite nanoparticles Ag@TiO_2_NPs.

The composite nanoparticles (Ag@TiO_2_NPs) were obtained by loading AgNPs particles on the surface of TiO_2_NPs using photocatalytic reduction, as shown in Figure 1. Briefly, P25-TiO2NPs (Sigma, United States) were ultrasonically dispersed in 5% alcohol solution to obtain a suspension of 1 mg/ml, and then the TiO_2_NPs suspension was mixed with 1 mg/ml of silver nitrate solution in equal volume. The AgNO_3_ & TiO_2_ suspensions were irradiated with UV light (UV light intensity = 10 mW/cm^2^, wavelength λ = 365 nm) at 2 mm liquid depth to obtain the Ag@TiO_2_NPs suspensions. Then the suspension was centrifuged, washed, and dried at 60°C, obtaining Ag@TiO_2_NPs. Photocatalytic reduction times were 1, 5, 20, and 60 s to obtain the composite nanoparticles labeled as “1#”, “2#”, “3#”, and “4#”. Samples storage away from light.

### 2.2 Preparation and Photo-Functionalization of Ag@TiO_2_NPs Coating

Ultrasonically resuspend Ag@TiO_2_NPs with 5% alcohol in the same volume as the Ag@TiO_2_NPs suspensions in Section 2.1. subsequently, 1 × 1 cm^2^ PU sheets were immersed in the suspension and deposited for 3 hours at room temperature to form a coating. Medical-grade silicone rubber (SR) catheters were peristaltic pump circulating with Ag@TiO_2_NPs suspension for 12 h to obtain the coating for the *ex-vivo* antithrombogenicity test in Section 2.5.2. After coating, samples were rinsed with RO water, dried under nitrogen, and stored away from light.

Photo-functionalization of the samples was achieved by UV irradiation (UV light intensity = 10 mW/cm^2^, wavelength λ = 365 nm) for 1 h.

The untreated coatings of TiO_2_, 1#, 2#, 3#, and 4# nanoparticles are labeled as “UNT-TiO_2_C”, “UNT-1#C”, “UNT-2#C”, “UNT-3#C”, “UNT-4#C”, and the corresponding UV photo-functionalized nanoparticles coatings are labeled as “UV-TiO_2_C”, " UV-1#C”, " UV-2#C”, " UV-3#C”, “UV-4#C”.

### 2.3 Physicochemical Characterization of Ag@TiO_2_NPs and Ag@TiO_2_NPs Coating

The microstructure and the element distribution of Ag@TiO_2_NPs were examined by field-emission transmission electron microscopy (TEM, JEM-2100F, JEOL, Japan) and the energy dispersive spectroscopy (EDS, JEM-2100F, JEOL, Japan).

Water contact angles (WCA) of Ag@TiO_2_NPs coatings were obtained by analyzing images using ImageJ software (National Institutes of Health, United States).

Photocatalytic degradation of methylene blue assay was performed to examine the photocatalytic activity of Ag@TiO_2_NPs coating. In brief, methylene blue powder was prepared as a 5 mg/L solution in deionized water. A sample was immersed in 1 ml of the solution, and UV irradiated (UV light intensity = 10 mW/cm^2^, wavelength λ = 365 nm) for 1, 3, and 5 h. 200 μL of the solution was collected at each time point, and the absorbance (A) at a wavelength of 664 nm was determined using a microplate reader (BIO-TEK Instruments, United States). The relationship between A and the degradation rate (G) was calculated as

$$\text{G}=\left[\left({\text{A}}_{\text{0}}-{\text{A}}_{\text{t}}\right)/{\text{A}}_{\text{0}}\right]\times 100\%$$ (1)

where A_0_ is the original absorbance of the undegraded methylene blue, and A_t_ is the absorbance value after t hours of degradation.

In the Ag^+^ release test, atomic absorption spectroscopy (AAS, TAS-990F, Beijing Purkinje General Instrument Co., Ltd., China) was performed to measure the release of Ag^+^ from different samples into phosphate-buffered saline (PBS; pH = 7.4). Briefly, PU samples coated with Ag@TiO_2_NPs were immersed in 1 ml of PBS in the dark. The PBS was collected and replaced with 1 ml of fresh PBS every 2 days. This process was repeated for a total of 14 days. All collected PBS solutions were analyzed for their content of Ag^+^, and an Ag^+^ time-release curve was plotted.

### 2.4 Antibacterial Assay

In this study, *Staphylococcus aureus* (*S. aureus*) and *Pseudomonas aeruginosa* (*P. aeruginosa*) were used to evaluate the antibacterial ability of the Ag@TiO_2_NPs coating. Briefly, *S. aureus* and *P. aeruginosa* obtained from Sichuan Provincial People’s Hospital were inoculated onto blood agar plates and cultivated in an incubator at 37°C. After the appearance of multiple colonies, *S. aureus* and *P. aeruginosa* were collected and dispersed in an F12 medium containing 10% fetal bovine serum (FBS, Sigma, United States). The density of *S. aureus* and *P. aeruginosa* was adjusted to 1 × 10^6^ colony forming units (CFU)/mL, and then 1 ml of bacterial suspension was added to the wells of a 24-well plate to soak the samples. After incubation at 37°C for 6 hours, the samples were washed three times and then transferred to a new 24-well plate containing a mixture of F12 FBS medium and cell counting kit-8(CCK-8, APExBIO Ltd., Houston, United States) agent. After 3 h of incubation, the activity of adherent bacteria was tested by detecting the absorbance of the medium at a wavelength of 450 nm using a microplate reader. The samples were then fixed with 2.5% glutaraldehyde for 12 h, after which they were dehydrated, and the adherent bacteria on the samples were observed and analyzed with an optical microscope (OM, DM4000M; Leica, Germany).

### 2.5 Evaluation of Hemocompatibility

#### 2.5.1 Platelet Static Adhesion Test

Fresh whole blood was drawn from human healthy adult volunteers and anticoagulated with citric acid dextrose (ACD) (blood to ACD ratio of 9:1), in compliance with the ethical standards of Southwest Jiaotong University. Then, the fresh ACD blood was centrifuged at 1,500 rpm for 15 min, and thus platelet-rich plasma (PRP) was obtained. The PU samples (1 × 1 cm^2^) deposited with the nanoparticles coatings were placed in 24-well cell culture plates and incubated with PRP (100 μL per sample) at 37°C for 1 hour under static conditions. After incubation, the samples were rinsed carefully three times with 0.9% saline to remove non-adherent platelets. Subsequently, the samples were fixed with 2.5% glutaraldehyde for 2 hours at room temperature. After typical rhodamine staining (Sigma, United States), the number of platelet adhesions in each sample was calculated by ImageJ software with six random fluorescence microscopy (DMRX, Leica, Germany) images (size = 400×). Furthermore, the morphology of the adhered platelets was observed under a scanning electron microscope (SEM, Quanta 200; FEI, Holland).

#### 2.5.2 Antithrombogenicity Test by Ex-Vivo Blood Circulation

All succeeding procedures were performed in compliance with the China Council on Animal Care and Southwest Jiaotong University Animal Use protocol, following all the ethical guidelines for experimental animals.

The establishment of *ex-vivo* blood circulation has been described elsewhere (Qiu et al., 2021). Thrombogenicity of the SR catheters was assessed using an *ex-vivo* arteriovenous (AV) shunt model in the rabbit. For the detail of the AV shunt model, a custom-built extracorporeal circulation (ECC) pipeline with three parallel channels was set up. The ECC pipeline was made of medical-grade polyvinyl chloride (PVC) tubing. Samples, including the uncoated SR, UNT-1#C, and UV-1#C catheters, were connected to the ECC system.

Rabbits (New Zealand white rabbits, 2.5 kg) were anesthetized by intravenous injection of sodium pentobarbital (30 mg/kg). The rabbit left carotid artery and the right external jugular vein were isolated through a midline neck incision. The AV custom-built extracorporeal circulation (ECC) was placed into position by cannulating the left carotid artery for ECC inflow and the right external jugular vein for ECC outflow (Figure 5A). The flow through the ECC was started by unclamping the arterial and venous sides of the ECC. Animals had no systemic anticoagulation throughout the experiment.

After 30min on ECC, the circuits were clamped, removed from the animal, rinsed with 0.9% saline (pH 7.4) gently, and drained. The cross-sections of the catheters were photographed for determination of the occlusion rates. Then, the residual thrombosis in the catheters was fixed in 2.5% glutaraldehyde solution overnight at room temperature and dehydrated. After weighing, samples undergo a micromorphological analysis by scanning electron microscopy (SEM, Quanta 200, FEI, Holland).

## 3 Results and Discussion

### 3.1 Characterization of Ag@TiO_2_NPs

**FIGURE 2:**
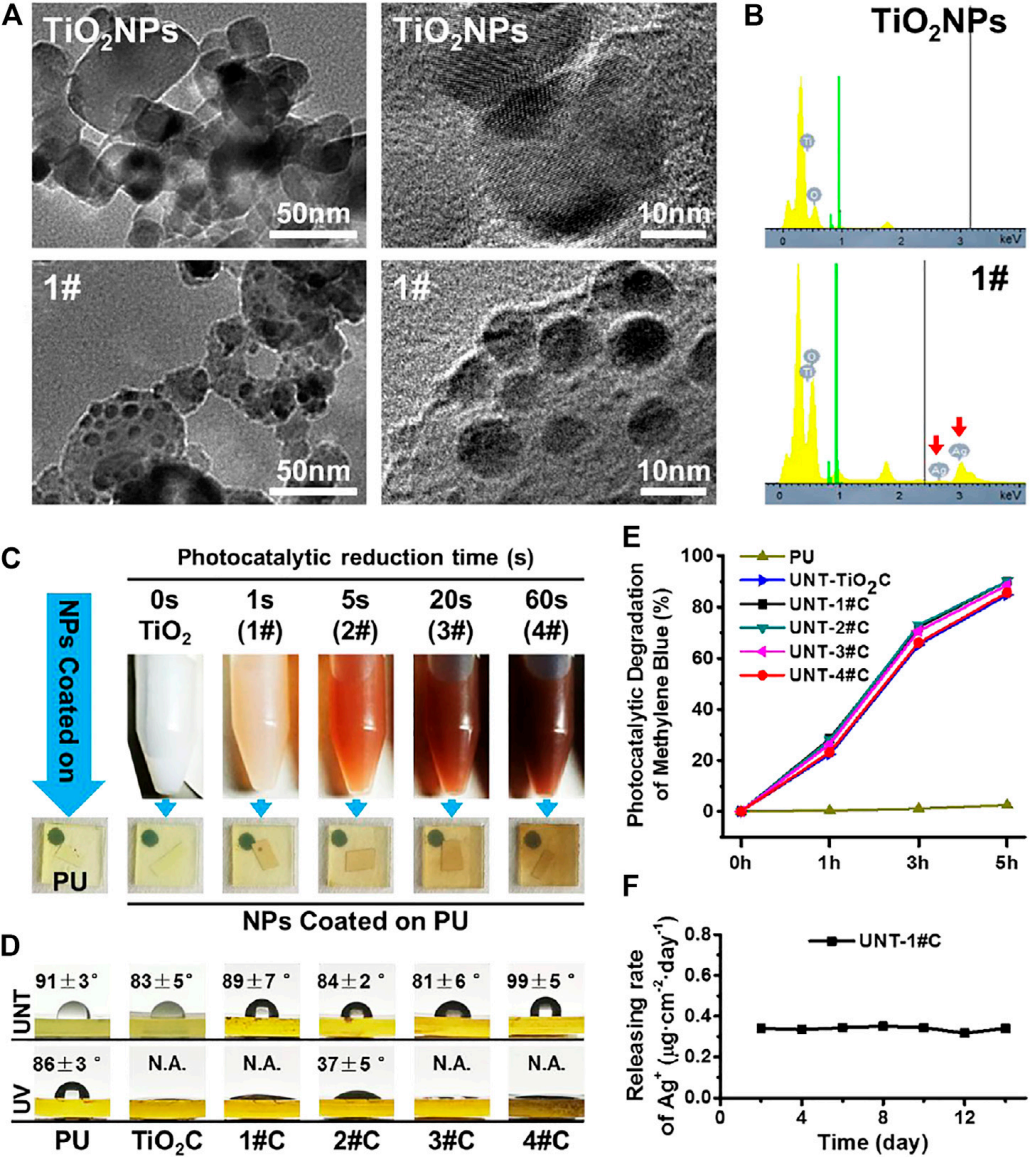
TEM microscopic images of TiO_2_NPs before and after photocatalytic reduction in silver nitrate solution **(A)** and the results of EDS elemental analysis **(B)**; photos of Ag@TiO_2_NPs suspensions and corresponding coating on PU sheets at different photocatalytic reduction times **(C)**; WCA of Ag@TiO_2_NPs coatings on PU sheets before and after photo-functionalization **(D)**; Untreated Ag@TiO_2_NPs coatings photocatalytic degrade of methylene blue **(E)**; Ag^+^ release from UNT-1# coating **(F)**.

To confirm that Ag@TiO_2_NPs were successfully prepared, TEM was used to characterize the microstructure of the nanoparticles, and EDS was used to perform elemental analysis of the nanoparticles. From the TEM results, additional spherical secondary structures with diameters of 5–10 nm were observed on the surface of 1# nanoparticles compared to TiO_2_NPs (Figure 2A). the signal of elemental Ag appeared in the EDS data for 1# nanoparticles (Figure 2B), which indicates that the spherical secondary structures appearing on the surface of 1# nanoparticles are AgNPs. These AgNPs should be the products of Ag^+^ reduction by electrons generated on the surface of TiO_2_NPs during the photocatalytic reduction process (Lu et al., 2013; Chen et al., 2021).

The results of TEM and EDS indicate that Ag@TiO_2_NPs were successfully prepared. Furthermore, the color of the suspensions of Ag@TiO_2_NPs deepened with the prolongation of the photocatalytic reduction time, which implied that the AgNPs content in Ag@TiO_2_NPs might increase with the photocatalytic reduction time prolongation (Lu et al., 2013). The color of the coatings deposited by Ag@TiO_2_NPs onto the PU sheets also showed the same trend (Figure 2C).

### 3.2 Characterization of Ag@TiO_2_NPs Coating

The photo-induced hydrophilicity of TiO_2_ has been reported in many pieces of literature (Carp et al., 2004). Excellent hydrophilicity is considered to facilitate biofouling resistance (He et al., 2021). Compared with the non-photo-functionalized UNT-Ag@TiO_2_NPs coatings, the photo-functionalized UV-Ag@TiO_2_NPs coatings showed a significant decrease in WCA and exhibited strong photo-induced hydrophilicity (Figure 2D), which means that photo-functionalized nanoparticle coatings may be somehow more anticoagulant and antibacterial than their counterparts in the group. Among all UV-Ag@TiO_2_NPs coatings, 2# coating had the highest water contact angle, perhaps related to the content of AgNPs on the surface of Ag@TiO_2_NPs, the exact mechanism of which needs to be further investigated.

The photocatalytic oxidation activity of TiO_2_ is another manifestation of the photocatalytic activity of TiO_2_, which is closely related to the mechanism of photo-induced anticoagulant properties of TiO_2_ (Chen et al., 2014; Chen et al., 2015). Although it has been reported in the literature that loading AgNPs on the surface of TiO_2_ can enhance the photocatalytic activity of TiO_2_ by forming Schottky energy barriers (Gomathi Devi and Kavitha, 2016). However, in this study, through the photocatalytic oxidation decomposition of methylene blue, we found that the loading of AgNPs did not significantly affect the photocatalytic oxidation activity of Ag@TiO_2_NPs coating, compared to TiO_2_NPs coating (Figure 2E).

The Ag^+^ release rate is closely related to AgNPs’ biosafety and antimicrobial properties (Marchioni et al., 2018). 1# nanoparticles were used to analyze their Ag^+^ release in PBS after coating on PU, and it was found that the Ag^+^ release rate of UNT-1#C was stable around 0.35 μg cm^−2^ day^−1^ (Figure 2F), which was considered to be safe (Liu et al., 2018).

### 3.3 Analysis of Antibacterial Property

**FIGURE 3:**
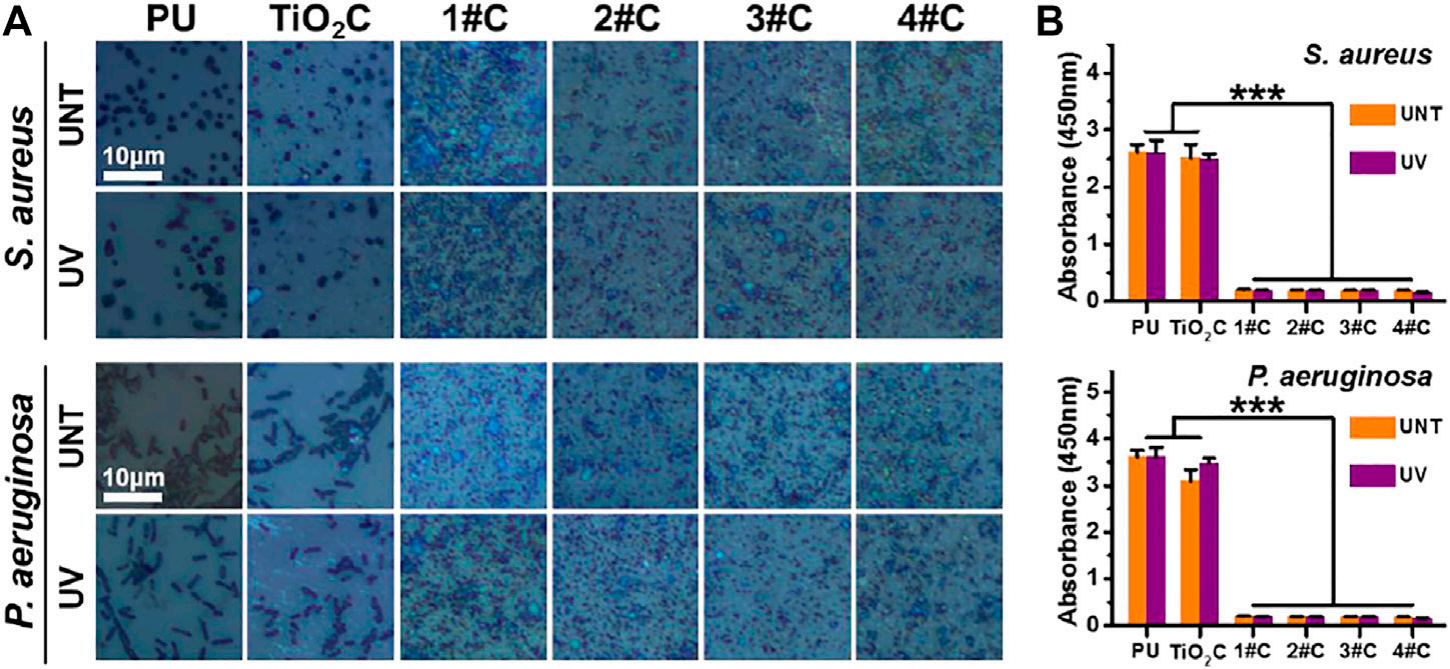
Antibacterial activity assay. Optical microscopy images of bacteria cultured on nanoparticles coatings before and after photo-functionalization **(A)** and bacterial activity measured by CCK-8 kit **(B)**.

Here, Gram-positive *Staphylococcus aureus* (*S. aureus*) and Gram-negative *Pseudomonas aeruginosa* (*P. aeruginosa*) were used to examine the broad-spectrum antimicrobial resistance of the Ag@TiO_2_NPs coatings. The results of optical microscopy showed that morphologically intact *S. aureus* and *P. aeruginosa* could be seen on the surface of all PU and TiO_2_NPs coatings, in contrast to all Ag@TiO_2_NPs coatings with a large amount of debris, probably necrotic bacterial fragments (Figure 3A).

The results of the cellular activity analysis (Figure 3B) were consistent with the observation under optical microscopy, where the bacterial activity on the surface of the TiO_2_NPs coating was similar to that of the PU. In contrast, all Ag@TiO_2_NPs coatings exhibited significant antimicrobial activity compared to the PU substrate and the TiO_2_NPs coating. Notably, there was no significant difference between the antimicrobial properties of UNT-Ag@TiO_2_NPs coatings and UV-Ag@TiO_2_NPs coatings, suggesting that the photo-functionalization treatment did not affect the AgNPs portion of Ag@TiO_2_NPs to perform the antimicrobial function. Another noteworthy point is that the antimicrobial performance of 1# coatings with the lowest AgNPs loading is comparable to that of 4# coaitngs with the highest AgNPs loading, indicating a powerful antimicrobial performance.

### 3.4 Analysis of Anticoagulant Property *in vitro*

**FIGURE 4:**
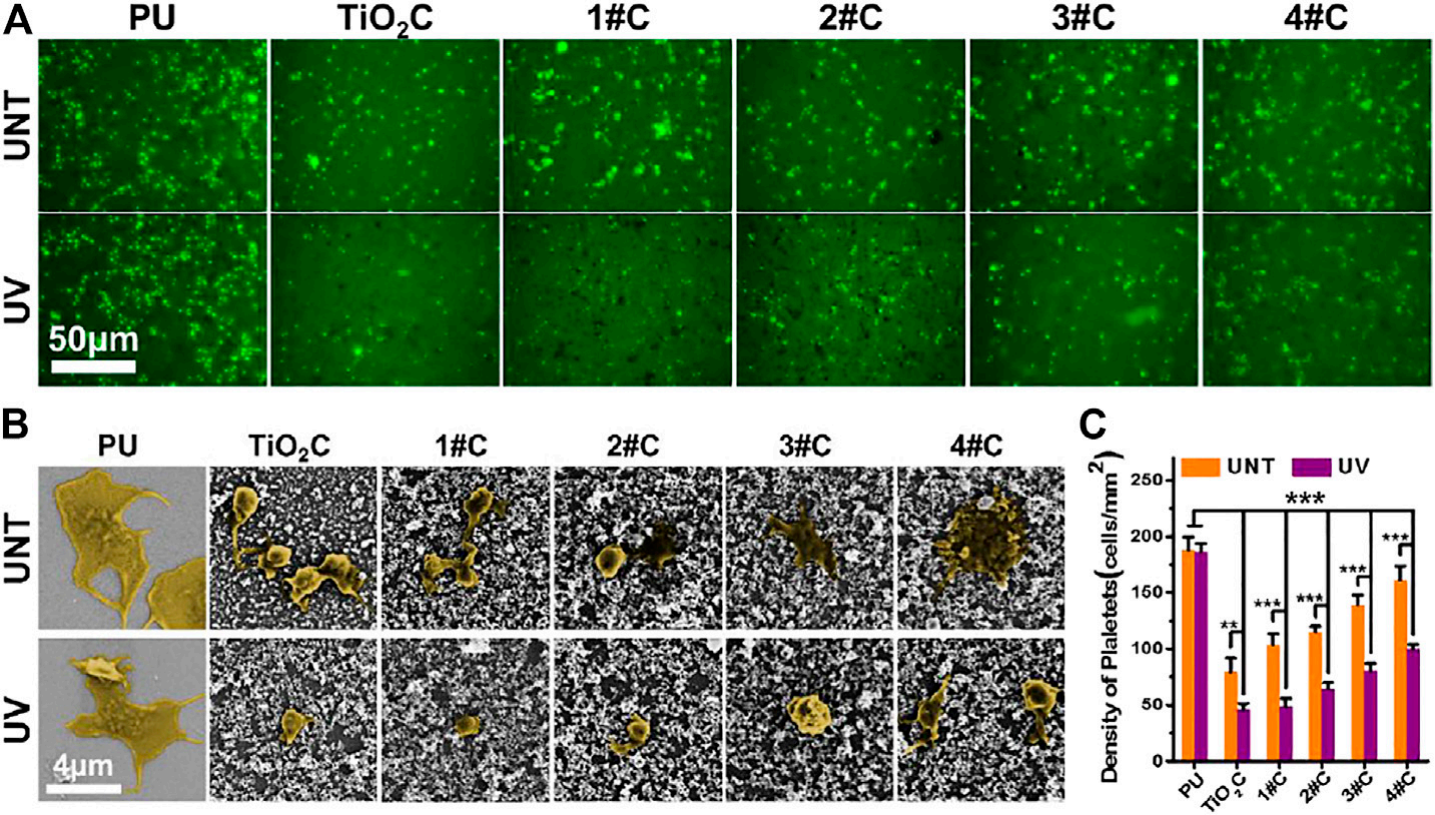
*In vitro* platelet adhesion assay. Fluorescence images **(A)**, SEM images **(B)**, and adhesion density **(C)** of adhered platelet on nanoparticles coatings.

*In vitro* platelet adhesion experiments were performed using deposited Ag@TiO_2_NPs coatings on PU sheets, for PU is widely used in various blood contact devices. The results of fluorescence images (Figure 4A) and adhesion density (Figure 4C) showed that all nanoparticles coatings significantly reduced the number of platelet adhesions after photo-functionalization compared with that before treatment. Besides, the platelet adhesions density on the photo-functionalized nanoparticle coatings was significantly lower than that of PU substrates. Notably, the platelet adhesion density of nanoparticles coatings tended to increase with the prolongation of photocatalytic reduction time in the composite nanoparticles preparation stage. Considering the issue of blood compatibility of AgNPs, this result is similar to the phenomenon that the color of Ag@TiO2NPs suspension deepens with increasing photocatalytic reduction time (Figure 2C), supporting the idea that the AgNPs content in Ag@TiO2NPs increases with increasing photocatalytic reduction time.The SEM results of platelet adhesion (Figure 4B) showed that the platelets on the PU substrate were in a partially spreading dendrites state, with the degree of spread much higher than that of platelets on the UV-photo-functionalized nanoparticles coating, which was between spherical to partially spread. In contrast, the degree of platelet spreading on all nanoparticles coatings showed a tendency to increase with increasing photocatalytic reduction time at the composite nanoparticles preparation stage. This trend is consistent with the results of platelet adhesion density, which could be similarly influenced by the content of AgNPs in the composite nanoparticles.

The results of platelet adhesion experiments suggested that the Ag@TiO_2_NPs coatings can acquire anti-platelet adhesion ability after photo-functionalization. However, this photo-induced anticoagulant properties of the UV-Ag@TiO_2_NPs may be weakened by increasing the content of AgNPs, this implies that the photo-induced anticoagulant properties of TiO_2_ have a limited or dose-dependent improvement on the hemocompatibility of AgNPs. Therefore, proper loading of AgNPs is essential to impart UV-Ag@TiO2NPs with excellent antimicrobial properties without causing deterioration of hemocompatibility.

Here, combined with the results of the antimicrobial experiments and *in vitro* platelet adhesion assays, the UV-1# sample with the lowest silver loading may be an ideal candidate capable of obtaining balanced antimicrobial and anticoagulant properties.

### 3.5 *Ex-vivo* Antithrombogenicity Test

**FIGURE 5:**
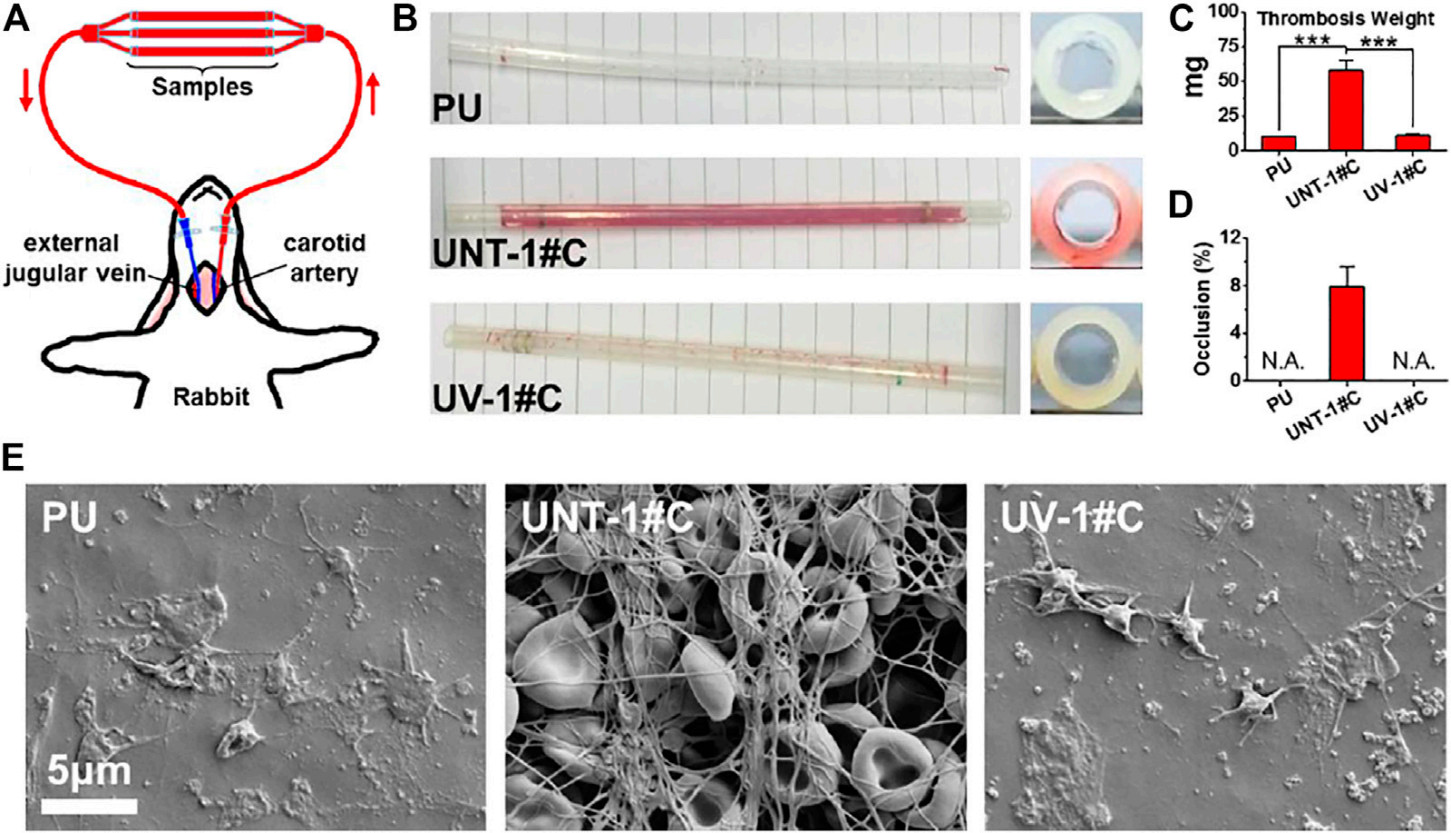
Schematic diagram of the *ex-vivo* blood circulation experiment **(A)**; photograph of the catheter after the experiment **(B)**; the weight of the thrombus formed in the catheter **(C)**; catheter occlusion rate **(D)**; microscopic image of the catheter lining by SEM **(E)**.

The Antithrombogenicity test by *ex-vivo* blood circulation provides a more comprehensive test of the anticoagulant capacity of the UV-Ag@TiO_2_NPs coating compared to the *in vitro* blood test. SR is a material commonly used for central venous catheters, which is a typical medical device that requires both anticoagulation and antimicrobial activity. Considering the *in vitro* platelet adhesion and Antibacterial Assay results, SR catheters deposited with 1# nanoparticles were selected for the *ex-vivo* blood circulation assay. After 30 min of *ex-vivo* blood circulation, the surface of the UNT-1# coating showed patches of sizable thrombus layer that blocked nearly 8% of the catheter lumen compared to the UV-1# coating with only a small amount of thrombus on the surface (Figures 5B,D). The analysis of the weight of the formed thrombus showed a significant increase in UNT-1# coating compared to both UV-1# coating and PU substrate (Figure 5C). At the same time, there was no significant difference in UV-1# coating compared to PU substrate.

SEM images of the inner wall of the catheter revealed that UNT-1# coating had fully initiated the coagulation mechanism, and the fibrinogen network captured a large number of red blood cells, forming a thrombus layer that completely covered the substrate. In contrast, the UV-1# coating and the PU substrate were similar, with only some platelets adhering to the surface (Figure 5E). It is worth noting that most of the erythrocytes in the thrombus layer remain in typical form, and combined with the data on the rate of silver ion release from the coating (Figure 2F), hemolysis may not be a problem that the coating would cause.

Here, UV-Ag@TiO_2_NPs coating did not show significantly better results than PU substrates in the *ex-vivo* blood assay as in the *in vitro* platelet adhesion assay. This may be due to the dynamic environment and more comprehensive blood composition in the *ex-vivo* blood circulation assay (Courtney et al., 1994). Nevertheless, the results of *in vitro* platelet adhesion and *ex-vivo* blood experiments were sufficient to suggest that loading AgNPs onto the surface of TiO_2_NPs and performing UV- Photo-functionalization treatment was an effective way to improve their hemocompatibility.

## 4 Conclusion

This study prepared a composite nanoparticles (Ag@TiO_2_NPs) by loading AgNPs onto TiO_2_NPs using a photocatalytic reduction method. Then, we utilized secondary UV irradiation to photo-functionalize Ag@TiO_2_NPs to improve the hemocompatibility of TiO_2_NPs and AgNPs. *In vitro* and *ex-vivo* experiments showed that such photo-functionalized UV-Ag@TiO_2_NPs coatings are endowed with excellent hemocompatibility. When UV-Ag@TiO_2_NPs were deposited onto the PU and SR to form a coating, they significantly inhibited platelet adhesion and activation, showing anticoagulant properties not inferior to those of medical-grade substrates. In addition, this UV-Ag@TiO_2_NPs coating exhibited excellent antibacterial properties against both Gram-positive and Gram-negative bacteria. Therefore, these UV-Ag@TiO_2_NPs could service the medical devices that require both anticoagulant and antibacterial properties, such as central venous catheters. More importantly, this UV- photo-functionalized modification strategy relying on TiO_2_ could provide a new idea to solve the problem of blood compatibility of functionalized metal nanoparticles similar to AgNPs.

## Data Availability

The original contributions presented in the study are included in the article/Supplementary Material, further inquiries can be directed to the corresponding authors.
